# Wider impacts of a 10-week community cooking skills program - Jamie’s Ministry of Food, Australia

**DOI:** 10.1186/1471-2458-14-1161

**Published:** 2014-12-12

**Authors:** Jessica Herbert, Anna Flego, Lisa Gibbs, Elizabeth Waters, Boyd Swinburn, John Reynolds, Marj Moodie

**Affiliations:** Deakin Health Economics, Faculty of Health, Deakin University, Melbourne, Victoria Australia; Jack Brockhoff Child Health and Wellbeing Program, Centre for Health Equity, Melbourne School of Population Health, The University of Melbourne, Melbourne, Victoria Australia; WHO Collaborating Centre for Obesity Prevention, Faculty of Health, Deakin University, Melbourne, Victoria Australia; School of Population Health, Faculty of Medical and Health Sciences, University of Auckland, Auckland, New Zealand; Deakin Biostatistics Unit, Faculty of Health, Deakin University, Melbourne, Victoria Australia

**Keywords:** Cooking skills, Healthy eating, Health promotion, Evaluation, Nutrition education

## Abstract

**Background:**

Jamie’s Ministry of Food (JMoF) Australia is a 10-week community-based cooking skills program which is primarily aimed at increasing cooking skills and confidence and the promotion of eating a more nutritious diet. However, it is likely that the program influences many pathways to behaviour change. This paper explores whether JMoF impacted on known precursors to healthy cooking and eating (such as attitudes, knowledge, beliefs, cooking enjoyment and satisfaction and food purchasing behaviour) and whether there are additional social and health benefits which arise from program participation.

**Methods:**

A mixed method, quasi-experimental longitudinal evaluation with a wait-list control was conducted. Intervention participants were measured using repeated questionnaires at three time points; before and after the program and at six-month follow-up. Control participants completed the questionnaire 10 weeks before their program and at program commencement. Quantitative analysis used a linear mixed model approach and generalised linear models for repeated measures using all available data. Qualitative methods involved 30-minute repeated semi-structured interviews with a purposively selected sample, analysed thematically.

**Results:**

Statistically significant differences between groups and over time were found for a reduction of take away/fast food weekly purchasing (*P* = 0.004), and increases in eating meals at the dinner table (*P = 0.01*), cooking satisfaction (*P = 0.01*), and the ability to prepare a meal in 30 minutes (*P < 0.001*) and from basics that was low in cost (*P < 0.001*). The qualitative findings supported the quantitative results. Repeat qualitative interviews with fifteen participants indicated increased confidence and skills gained from the program to prepare meals from scratch as well as increases in family involvement in cooking and meal times at home.

**Conclusions:**

Jamie’s Ministry of Food, Australia resulted in improvements in participants’ food and cooking attitudes and knowledge, food purchasing behaviours and social interactions within the home environment, which were sustained six months after the program.

**Trial registration:**

Australian and New Zealand Trial registration number: ACTRN12611001209987.

**Electronic supplementary material:**

The online version of this article (doi:10.1186/1471-2458-14-1161) contains supplementary material, which is available to authorized users.

## Background

There is a common discourse about a lack of home cooking and food skills in westernized societies today. Factors contributing to this lack of cooking and decline in skills include competing time demands, busy schedules, daily stressors, lack of cooking knowledge and confidence and an increased reliance on prepared food [1–3]. This problem has raised interest from both the media and health promotion sectors, resulting in an increased number of not-for-profit community-based cooking skills programs, both in Australia and internationally [4–9].

There are many factors that influence whether individuals and families will cook and eat healthy meals. These include cooking and eating knowledge, attitudes and beliefs, and enjoyment and satisfaction with the cooking experience. The perceived cost of healthy food can be a barrier to a healthy diet [5, 10]. Healthy food choices are influenced by food purchasing behaviours, particularly around vegetable purchasing. Results from a Brisbane food study in Australia found that low socio-economic positioned (SEP) groups purchased fewer types of fruit and vegetables, fewer foods high in fibre and low in fat compared to high SEP groups [11]. Those with low education and income levels were less likely to comply with dietary guidelines in terms of fruit and vegetable consumption as reflected by their fruit and vegetable purchasing [11]. In addition, increased confidence to prepare vegetables has been found to be related to purchasing a greater variety of vegetables and more often [12]. One could assume that by improving cooking confidence around fruit and vegetable preparation this may impact on food purchasing attitudes and behaviours.

Changes to traditional family meal patterns have been reported. Busy schedules and competing priorities impact on the frequency of family meals [13, 14]. The traditional family meal around the dinner table with food prepared from fresh ingredients or ‘from scratch’ is no longer a cultural norm [15]. These factors are cause for concern because the frequency of family meals has been found to have a protective health factor through improved dietary outcomes [16]. There is evidence to suggest that children of families who always eat dinner together at the table consume more fruit and vegetables compared to children of families who never eat together [17]. Eating meals in front of the television is associated with negative health impacts such as higher body mass index, particularly in older children [18]. In a nationwide survey of 1,011 Australians in 2008, 60% of respondents reported that the television was always or often on during meal time [19].

Jamie’s Ministry of Food (JMoF) was originally developed by celebrity chef Jamie Oliver in the United Kingdom (UK) [20], and in recent years brought to Australia and adapted to an Australian setting. The program is a community-based cooking skills program consisting of ten weekly 90-minute classes, aimed at getting people of all ages and backgrounds cooking simple, fresh, healthy food quickly and easily [21]. The JMoF program focuses on building positive attitudes and increasing knowledge, skills and self-efficacy related to healthy eating, food and cooking. It was brought to Australia by a not-for-profit, health promotion organisation, The Good Foundation (TGF), and The Good Guys, a major Australian electrical goods retailer, in partnership with Jamie Oliver. Ipswich in Queensland was the first Australian centre to open and commenced operation in April 2011. The Ipswich Centre is primarily funded by philanthropist Mr Andrew Muir (owner of The Good Guys) and Queensland State Government, as well as local partners. Ipswich was intentionally selected as a target site given its low SEP [22] and increasing levels of overweight and obesity [23] within the population. This program has been evaluated both in terms of the primary aims of increasing cooking confidence and vegetable consumption (*reported elsewhere* [24]) and its wider dietary, health and psychosocial impacts.

A program logic model was developed to explore the pathways that might impact on behaviour change in terms of cooking and food behaviours [25]. It is understood that changes in attitude, beliefs and self-efficacy are important pre-cursors to behaviour change [26]. There is evidence to suggest that improved cooking confidence may impact on cooking behaviours [9] as well as healthy diets [12], however the evidence around other effects on cooking behaviours is less strong and warrants more research [27]. This paper explores the 10-week program’s impacts on the other influences on cooking and eating (specifically, cooking and healthy eating attitudes, beliefs and knowledge, food purchasing behaviours, cooking enjoyment and satisfaction, and social and health benefits).

## Methods

The evaluation methods are detailed in Flego et al. [25], but are briefly summarised here. A longitudinal mixed methods design was adopted for the evaluation. The quantitative component measured changes in participant skills, knowledge, attitudes, and food purchasing behaviours, cooking enjoyment and satisfaction, social connectedness around food and health effects as a result of the JMoF program. The qualitative component aimed to provide a deeper understanding of participant experiences of the program and to explore the barriers and facilitators to cooking. The qualitative and quantitative studies ran concurrently and results were analysed separately before being combined.

### Quantitative study

The quantitative component used a quasi-experimental design with a wait-list control group. Intervention participants were measured at program commencement (T1), at program completion (T2) approximately 10 weeks after commencement, and six months later (T3) approximately six months after program completion. The control group (participants who registered for the program ten weeks in advance) were measured ten weeks prior to (T1) and at program commencement (T2). Controls were not measured at six month follow-up (T3) because it was deemed impractical to make participants wait six months to attend the program. At each time point, participants completed a 15-minute self-administered questionnaire designed to elicit self-reported information around key program domains.

Recruitment to the evaluation was restricted to participants over the age of 18 years. The study was designed to detect a change in mean daily vegetable intake (primary outcome) of 0.5 serves per day, from a baseline of 2.5 serves per day, with 80% power using a two-sided test at the 5% significance level – this required a minimum of 140 participants in each group [25]. Recruitment to the study continued until the sample reached the target of 140 participants in each group at T2. Statistical analyses were based on the set of individuals who registered for the program, responded to an invitation to participate in the evaluation and subsequently completed the baseline questionnaire.

Multilevel mixed linear models were used to analyse all continuous-scale, repeated measures data [28, 29]. Results from these analyses are presented in the form of predicted means, recovered from the fitted mixed linear model, and their associated standard errors (SE). Differences in these predicted means over time and within each participant group are also presented. Responses to questions on nutritional knowledge were dichotomised into correct or incorrect responses. The proportions of correct responses in subgroups are presented and comparisons of subgroups were based on generalized linear models, fitted using the method of generalized estimating equations (GEE) which allows for longitudinal binary data [29].

Two analyses of each outcome variable were conducted: (i) comparisons between groups of their changes over time from T1 to T2 (equivalent to testing for a time by group interaction), and, (ii) comparisons of the three time points (T1, T2 and T3) within the intervention group. Each model-based analysis was also re-run adjusting for covariates (such as age, gender, and employment status) when the covariates exhibited baseline differences (i.e. differences at T1) between the control and intervention groups. Analyses was performed using STATA (version 12.0) [30]. Results were considered significant if *P < 0.05*.

### Qualitative study

A selected number of JMoF program participants were approached to be involved in the qualitative study. Purposive sampling was employed, utilising maximum variation [31] to ensure a diverse group of participants in terms of socio-demographic characteristics such as socio-economic status, age, gender, family structure, and cooking confidence level.

Recruitment of participants occurred via two ways [25]. Firstly, in the quantitative questionnaire, participants were asked about their willingness to be contacted for a future interview. Secondly, purposive sampling was undertaken by the researcher whilst observing groups in their first week of the program.

**Figure 1:**
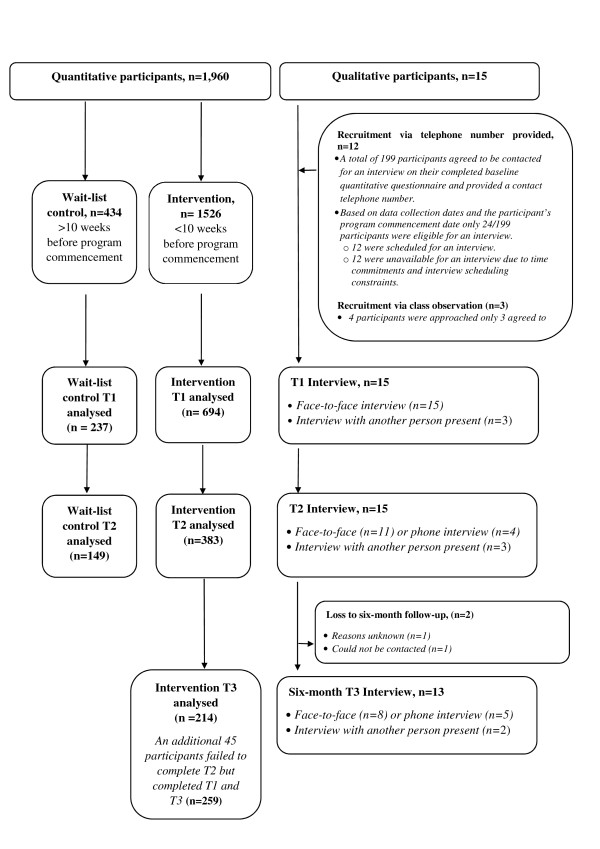
**Jamie’s ministry of food mixed methods evaluation, quantitative and qualitative participation.** The quantitative component began in November 2011 – December 2013, the qualitative evaluation began in August 2012 – July 2013.

The interviews were conducted face-to-face or by phone by a trained researcher, Figure 1. The first interview was conducted before the program had commenced or no greater than two weeks into the program. Repeat interviews with the same people were conducted at program completion, and at six months post completion.

The three repeat semi-structured interviews were approximately 30–40 minutes long and were conducted in a public location in Ipswich. The interviews explored participant expectations and experiences of the program and reflections on the impact of the program on their attitudes and behaviours, described in more detail elsewhere [25]. Participants were given a $15 supermarket gift voucher at the end of each interview in appreciation of their time.

All interviews were recorded and transcribed verbatim, with participant consent. The data was managed with the assistance of a qualitative software package NVivo 9 (NVivo 9 [program]: QSR International Pty Ltd 2011). Transcripts and data memos were coded, then categorised to identify themes and emerging patterns. A second researcher independently generated codes on a sub-sample of transcripts and any differences between codes were discussed [32, 33]. Analysis was conducted concurrently with data collection allowing for ongoing clarification of emerging findings [32]. Further analysis of the themes was conducted in comparison with relevant literature to determine alignment with existing evidence [32, 33].

### Ethics

Ethics approval was received from Deakin University Human Research Ethics Committee (HEAG-H 117_11). Informed consent was received for both quantitative and qualitative components of the study [25].

## Results

### Quantitative results

#### Participants

Figure 1 provides a summary of the participants involved in both the quantitative and qualitative components of the evaluation. In the quantitative analysis, a total of 694 intervention participants completed T1 measurements, 383 at T2 and 259 at T3. In the wait-list control group, 237 participants completed the survey at T1and 149 at T2. Further details of participant dropout rates are presented elsewhere, as is a detailed description of the demographic profile [24]. At baseline, most participants spoke English at home, were female, resided within the Ipswich district, and were in full or part-time employment. There were three significant differences between the control and intervention groups - the control group was younger (64.3% aged below 50 years compared to 55.6% in the intervention group and median ages of 48 and 46 years in the control and intervention groups respectively); was comprised of fewer males (12.8% compared to 22.6% males in the intervention group) and was more likely to have participants in full employment (34.7% compared to 26.4% in the intervention group).

#### Food purchasing behaviours and attitudes

There was a statistically significant decrease in total weekly take away/fast food expenditure in the intervention group (*P < 0.001*) but not in the control group between T1 and T2. This was the only food purchasing behaviour to show a statistically significant group by time interaction (*P = 0.004*) (Table 1). However total fruit and vegetable expenditure did significantly increase by a mean AUD2.50 over time in intervention group between T1 and T2 (*P < 0.001*). There was a statistically significant increase in the numbers believing that they could prepare a meal from basics that was low in price between T1 and T2 in the intervention group (*P < 0.001*) but not the control. There was also a small significant increase in knowledge around cost of fruit and vegetables being cheaper when in season in the intervention group (*P < 0.001*) but not in the control.

**Table 1:** **Secondary outcome measures by group at baseline and follow up** ^**1**^

Outcome measure	Intervention			Control			Difference between groups in changes over time (interaction effect) ^3^ ***P value***
	Baseline (T1) mean (S.E) ^2^	Follow up (T2) mean (S.E)	Change from baseline (T2-T1) mean (S.E) ***P value***	Baseline (T1) mean (S.E) ^2^	Follow up (T2) mean (S.E)	Change from baseline(T2-T1) mean (S.E) ***P value***	
**Food purchasing behaviours and attitudes**							
*Total weekly food and drink expenditure (AUD)* ^4^	137.16 (2.72)	135.60 (3.15)	−1.56 (2.46) P = 0.53	147.34 (4.68)	151.68 (5.20)	4.33 (3.96) P = 0.27	P = 0.21
*Total weekly fruit and veg expenditure (AUD)* ^4^	20.77 (0.61)	23.28 (0.73)	2.50 (0.63) P < 0.001	21.70 (1.06)	22.24 (1.20)	0.53 (1.01) P = 0.60	P = 0.10
*Total weekly take away/fast food expenditure (AUD)* ^4^	13.17 (0.59)	9.86 (0.69)	−3.31 (0.55) P < 0.001	12.395 (1.01)	12.05 (1.13)	−0.34 (0.87) P = 0.70	P = 0.004
*I can prepare a meal from basics that is low in price* ^5^	2.99 (0.03)	3.41 (0.04)	0.41 (0.04) P < 0.001	3.00 (0.05)	2.97 (0.06)	−0.02 (0.06) P = 0.71	P <0.001
*Buying more fruit/vegetables would not be difficult on my budget* ^*5a*^	2.85 (0.03)	2.93 (0.04)	0.08 (0.04) P = 0.06	2.85 (0.06)	2.89 (0.07)	0.04 (0.07) P = 0.59	P = 0.60
*Fruit and vegetables are cheaper when they are in season* ^*5*^	3.42 (0.02)	3.62 (0.03)	0.21 (0.03) P < 0.001	3.43 (0.04)	3.50 (0.05)	0.07 (0.06) P = 0.21	P = 0.04
**Cooking and healthy eating knowledge, attitudes beliefs and behaviours**							
*I can put together a healthy meal from scratch in 30 minutes* ^*5*^	2.85 (0.031)	3.30 (0.04)	0.45 (0.04) P < 0.001	2.85 (0.05)	2.89 (0.06)	0.03 (0.06) P = 0.61	P <0.001
*I find it easy to change my eating habits* ^*5*^	2.52 (0.03)	2.71 (0.04)	0.19 (0.04) P < 0.001	2.52 (0.05)	2.53 (0.06)	0.01 (0.06) P = 0.82	P = 0.02
*Vegetables can be tasty foods* ^*5*^	3.54 (0.02)	3.69 (0.03)	0.15 (0.03) P < 0.001	3.53 (0.04)	3.51 (0.05)	−0.02 (0.05) P = 0.74	P = 0.01
*I eat enough fruit and vegetables* ^*5*^	2.66 (0.03)	3.00 (0.04)	0.34 (0.04) P < 0.001	2.66 (0.06)	2.68 (0.07)	0.02 (0.06) P = 0.71	P <0.001
*My lifestyle does not Prevent me eating a healthy diet* ^*5a*^	3.11 (0.03)	3.33 (0.04)	0.22 (0.04) P < 0.001	3.04 (0.05)	3.12 (0.06)	0.08 (0.06) P = 0.17	P = 0.07
**Cooking enjoyment and satisfaction**							
*I enjoy cooking* ^*5*^	3.05 (0.03)	3.33 (0.04)	0.28 (0.03) P < 0.001	3.12 (0.05)	3.17 (0.06)	0.06 (0.05) P = 0.28	P = 0.001
*I get a lot of satisfaction from cooking my meals* ^*5*^	2.96 (0.03)	3.31 (0.04)	0.35 (0.03) P < 0.001	3.02 (0.05)	3.05 (0.06)	0.03 (0.05) P = 0.60	P <0.001
*I enjoy cooking for others* ^*5*^	3.01 (0.03)	3.27 (0.04)	0.26 (0.03) P < 0.001	3.09 (0.06)	3.16 (0.07)	0.07 (0.06) P = 0.22	P = 0.004
I *enjoy eating a meal with others* ^*5*^	3.51 (0.02)	3.60 (0.03)	0.09 (0.03) P = 0.01	3.47 (0.39)	3.55 (0.05)	0.07 (0.05) P = 0.16	P = 0.81
**Social eating**							
*Frequency of eating together at home with others* ^*6*^	3.94 (0.07)	4.20 (0.08)	0.24 (0.07) P < 0.001	3.97 (0.11)	4.02 (0.13)	0.06 (0.11) P = 0.61	P = 0.13
*Frequency of eating dinner in front of the television* ^*6*^	2.69 (0.08)	2.50 (0.09)	−0.19 (0.07) P = 0.01	2.51 (0.14)	2.52 (0.15)	0.00 (0.11) P = 0.99	P = 0.17
*Frequency of eating dinner at a dinner table* ^*6*^	3.12 (0.08)	3.40 (0.09)	0.29 (0.06) P < 0.001	3.11 (0.13)	3.09 (0.14)	−0.02(0.10) P = 0.86	P = 0.01
**Health and emotional well-being**							
*Global self-esteem score* ^*7*^	20.88 (0.22)	22.60 (0.25)	1.73 (0.20) P <0.001	20.46 (0.37)	21.02 (0.42)	0.56 (0.32) P = 0.09	P = 0.002
*General Health* ^*8*^	2.77 (0.04)	3.11 (0.04)	0.34 (0.04) P <0.001	2.80 (0.06)	2.86 (0.07)	0.06 (0.06) P = 0.34	P <0.001
*Body Mass Index (BMI)*	28.86 (0.27)	28.78 (0.28)	−0.09 (0.13) P = 0.49	29.71 (0.46)	29.70 (0.47)	−0.02 (0.20) P = 0.94	P = 0.76

^1^Outcomes within each group and over time were determined by a mixed linear model for repeated measures using all available data at each time point using STATA (version 12.0).

^2^Baseline values were not significantly different between groups (independent t tests). ^3^A significant group and time interaction effect denotes that the response over time differed between groups (*P = 0.05*). ^4^Expenditure data was collected in Australian dollars (AUD) on a 7-point scale which was analyse by its midpoints. ^5^Mean predicted score indicating level of agreement with statement from a Likert Scale (*1 = strongly disagree, 2 = somewhat disagree, 3 = somewhat agree, 4 = strongly agree*), ^a^Score assignment was reversed. ^6^Mean frequency for a typical week was collected on a 6 or 7-point scale which was analyse by its midpoint, with the maximum category being five or more times per week. ^7^Rosenberg’s global self-esteem score *(Low self-esteem = 0-14, Normal self-esteem = 15-25, High self-esteem = 16-30).* ^8^Perceived general health (*poor = 1, fair = 2, good = 3, very good = 4, excellent = 5).*

When the analysis was restricted to the intervention group across the three time points (Table 2), overall expenditure on food and drink did not change, but there were significant increases between T1 and T3 in fruit and vegetable expenditure, preparing low cost meal from scratch, attitudes around buying more fruit and vegetables and the knowledge that fruit and vegetables are cheaper when in season. There was also a significant decrease in take away/fast food expenditure between T1 and T3 (*P < 0.001*).

**Table 2:** **Secondary outcome measures for the intervention group only at baseline (T1), post intervention (T2) and 6 months follow up (T3)** ^**1**^

Outcome measure	Baseline (T1) mean (S.E)	Follow up (T2) mean (S.E)	6 months post intervention follow up (T3) mean (S.E)	Change from baseline (T2-T1) mean (S.E) P value	Change from baseline (T3-T1) mean (S.E) P value	Change from baseline (T3-T2) mean (S.E) P value	Overall effect of change over time ***P value*** ^2^
**Food purchasing behaviours and attitudes**							
*Total weekly food and drink expenditure (AUD)* ^3^	137.13 (2.65)	135.21 (3.08)	137.28 (3.42)	−1.93(2.48) P = 0.44	0.15 (2.90) P = 0.96	2.08 (3.08) P = 0.50	P = 0.70
*Total weekly fruit and veg expenditure (AUD)* ^3^	20.77 (0.62)	23.25 (0.74)	23.64 (0.83)	2.48 (0.65) P < 0.001	2.86 (0.76) P < 0.001	0.39 (0.81) P = 0.63	P <0.001
*Total weekly take away/fast food expenditure (AUD)* ^3^	13.19 (0.59)	9.85 (0.68)	9.14 (0.76)	−3.34 (0.54) P < 0.001	−4.05 (0.63) P < 0.001	−0.71 (0.68) P = 0.29	P <0.001
*I can Prepare a meal from basics that is low in Price* ^4^	2.99 (0.03)	3.41 (0.04)	3.42 (0.04)	0.42 (0.04) P < 0.001	0.43 (0.05) P < 0.001	0.01 (0.05) P = 0.79	P <0.001
*Buying more fruit/vegetables would not be difficult on my budget* ^*4a*^	2.85 (0.03)	2.93 (0.04)	2.97 (0.05)	0.08 (0.05) P = 0.09	0.11 (0.05) P = 0.03	0.04 (0.06) P = 0.52	P = 0.06
*Fruit and vegetables are cheaper when they are in season* ^*4*^	3.42 (0.02)	3.62 (0.03)	3.66 (0.04)	0.21 (0.04) P < 0.001	0.24 (0.04) P < 0.001	0.04 (0.04) P = 0.41	P <0.001
**Cooking and healthy eating knowledge, attitudes beliefs and behaviours**							
*I can Put together a healthy meal from scratch in 30 minutes* ^*4*^	2.85 (0.03)	3.29 (0.04)	3.31 (0.05)	0.44 (0.04) P < 0.001	0.46 (0.05) P < 0.001	0.02 (0.06) P = 0.67	P <0.001
*I find it easy to change my eating habits* ^*4*^	2.52 (0.03)	2.71 (0.04)	2.70 (0.04)	0.17 (0.04) P < 0.001	0.18 (0.05) P < 0.001	0.00 (0.05) P = 0.94	P <0.001
*Vegetables can be tasty foods* ^*4*^	3.54 (0.02)	3.69 (0.03)	3.69 (0.04)	0.15 (0.03) P = 0.001	0.15 (0.04) P < 0.001	0.00 (0.04) P = 0.97	P <0.001
*I eat enough fruit and vegetables* ^*4*^	2.66 (0.03)	3.00 (0.04)	3.05 (0.05)	0.34 (0.04) P < 0.001	0.39 (0.05) P = 0.001	0.06 (0.05) P = 0.26	P <0.001
*My lifestyle does not Prevent me eating a healthy diet* ^*4a*^	3.11 (0.03)	3.32 (0.04)	3.29 (0.05)	0.21 (0.04) P < 0.001	0.18 (0.05) P < 0.001	−0.03 (0.05) P = 0.55	P <0.001
**Cooking enjoyment and satisfaction**							
*I enjoy cooking* ^*4*^	3.05 (0.03)	3.32 (0.04)	3.28 (0.04)	0.27 (0.03) P < 0.001	0.23 (0.04) P < 0.001	−0.04 (0.04) P = 0.31	P <0.001
*I get a lot of satisfaction from cooking my meals* ^*4*^	2.96 (0.03)	3.31 (0.04)	3.29 (0.04)	0.35 (0.04) P < 0.001	0.33 (0.04) P < 0.001	−0.02 (0.04) P = 0.72	P <0.001
*I enjoy cooking for others* ^*4*^	3.01 (0.03)	3.26 (0.04)	3.18 (0.05)	0.25 (0.04) P < 0.001	0.18 (0.04) P < 0.001	−0.08 (0.05) P = 0.11	P <0.001
*I enjoy eating a meal with others* ^*4*^	3.51 (0.02)	3.60 (0.03)	3.61 (0.03)	0.09 (0.03) P = 0.01	0.10 (0.04) P = 0.01	0.01 (0.04) P = 0.77	P = 0.003
**Social eating**							
*Frequency of eating together at home with others* ^*5*^	3.92 (0.07)	4.17 (0.08)	4.20 (0.09)	0.25 (0.07) P < 0.001	0.28 (0.09) P < 0.001	0.04 (0.09) P = 0.69	P <0.001
*Frequency of eating dinner in front of the television* ^*5*^	2.69 (0.08)	2.50 (0.09)	2.46 (0.10)	−0.19 (0.07) P = 0.01	−0.23 (0.08) P = 0.01	−0.04 (0.09) P = 0.66	P = 0.01
*Frequency of eating dinner at a dinner table* ^*5*^	3.12 (0.08)	3.40 (0.09)	3.37 (0.10)	0.28 (0.65) P < 0.001	0.25 (0.08) P = 0.001	−0.02 (0.08) P = 0.76	P <0.001
**Health and emotional well-being**							
*Global self-esteem score* ^*6*^	20.88 (0.22)	22.61 (0.25)	22.92 (0.28)	1.73 (0.21) P < 0.001	2.04 (0.25) P < 0.001	0.31 (0.26) P = 0.24	P <0.001
*General Health* ^*7*^	2.77 (0.04)	3.11 (0.05)	3.24 (0.05)	0.34 (0.04) P < 0.001	0.47 (0.05) P < 0.001	0.13 (0.05) P = 0.01	P <0.001
*Body Mass Index (BMI)*	28.86 (0.27)	28.79 (0.28)	28.94 (0.29)	−0.07 (0.14) P = 0.61	0.08 (0.16) P = 0.65	0.15 (0.17) P = 0.39	P = 0.68

^1^Outcomes within each group and over time were determined by a mixed linear model for repeated measures using all available data at each time Point using STATA (version 12.0).

^2^A significant group and time interaction effect denotes that the response over time differed between groups (*P < 0.05*). ^3^Expenditure data was collected in Australian dollars (AUD) on a 7-Point scale which was analyse by its midpoints. ^4^Mean Predicted score indicating level of agreement with statement from a Likert Scale (*1 = strongly disagree, 2 = somewhat disagree, 3 = somewhat agree, 4 = strongly agree*), ^a^Score assignment was reversed. ^5^Mean frequency for a typical week was collected on a 6 or 7-Point scale which was analyse by its midpoint, with the maximum category being five or more times Per week. ^6^Rosenberg’s global self-esteem score *(Low self-esteem = 0–14, Normal self-esteem = 15-25 and High self-esteem = 16-30).* ^7^Perceived general health (*Poor = 1, fair = 2, good = 3, very good = 4, excellent = 5*).

#### Cooking and healthy eating knowledge, attitudes, beliefs

The belief that participants could prepare a healthy meal from scratch in 30 minutes increased significantly in the intervention group but not in the control group between T1 and T2. A statistically significant group by time interaction (*P < 0.001*) also indicated a difference between groups in their changes and over time (Table 1). Table 1 also indicates statistically significant group by time interactions in beliefs around ease of changing eating habits (*P = 0.02*), vegetables being tasty (*P = 0.01*), and eating enough fruit and vegetables (*P < 0.001*). In the intervention group only analyses of the three time points, all attitudes and knowledge around cooking and healthy eating were significantly sustained from baseline (T1) to 6 months post program (T3) (Table 2).

Participants were asked to select the healthiest option from a range of food choices to test their knowledge around salt, sugar and fat content (results presented in text and not shown in a table). Based on the test for a group by time interaction in the GEE analysis, there was a significant increase in salt knowledge in the intervention group with 89.2% of participants indicating the correct answer at baseline and 94.75% at T2 and no change in the control group between T1 and T2 (91.45% and 90.41%; *P =* 0.04. Sugar knowledge increased in the intervention group between T1 (87.1%) and T2 (92.2%), and this was significantly different (*P = 0.02*) from the change over time in the control group between T1 and T2 (88.94% and 86.49% respectively). Changes in fat knowledge over time were also significantly different between the control and intervention groups (*P = 0.03*), with a larger increase in the control group (67.7% at T1, 79.9% at T2) compared to the intervention group (71.0% at T1, 74.5% at T2). When the analysis was restricted to participants in the intervention group, between T2 and T3, there was a significant increase (*P = 0.02*) in salt knowledge (93.4% at T3). But there was a significant (*P = 0.001*) decrease in sugar knowledge (90.3% at T3). Lastly, knowledge around fat appeared to decrease at T3 (69.4%) but not significantly (*P =* 0.42).

#### Cooking enjoyment and satisfaction

There were small but statistically significant differences in the increases over time and between groups in cooking enjoyment (*P = 0.001*), cooking satisfaction (*P < 0.001*) and cooking for others (*P = 0.004*) (Table 1). In the intervention group, all improvements in the level of cooking enjoyment and satisfaction were sustained at T3 (Table 2).

#### Social eating

Weekly frequency of eating dinner at the dinner table increased significantly in the intervention group (*P < 0.001*) but not in the control group. The overall group by time interaction was statistically significant (*P = 0.01*) (Table 1). The improvements in the intervention group in terms of behaviours around meal location and eating with others were statistically significant over time (Table 2). Between baseline (T1) and six months after the program (T3) there were significant increases in frequency of eating with others (*P < 0.001*) and decreased eating in front of the television (*P = 0.01*).

#### Health and emotional well-being

The JMoF program did not impact on participants’ self-reported BMI between groups and over time (Table 1). There was however a significant group by time interaction in both self-reported general health (*P < 0.001*) and self-esteem (*P = 0.002*). There was a statistically significant increase in global self-esteem in the intervention group (*P < 0.001*) but not in the control group. There was also a significant increase in general health in the intervention group (*P < 0.001*), but not in the control group. The improvement in general health continued to T3 and the improvement in self-esteem was maintained at T3 (Table 2).

#### Adjusted analyses of outcomes

Analyses of each outcome adjusted for age, gender and employment separately, then all together, to account for differences in composition between the non-randomized intervention and control groups, showed small differences in the predicted group by time means however pairwise comparisons remained similar to those in the results of the unadjusted analyses (results not shown, see [Additional file 1]).

### Qualitative results

#### Participants

Fifteen participants participated in the qualitative study. All completed T1 and T2 interviews, whilst 13 competed T3 interviews (with 2 lost to follow up) (Figure 1). The interviewees represented a heterogeneous cross-section of people from various stages of life. They varied in age (from 21–69 years old), household characteristics and levels of food preparation, responsibility and confidence, i.e. key factors which impacted on their home cooking and their willingness to learn and ability to make changes. There were more females interviewed than males, which reflects overall program enrolment. Two participants were interviewed together and one participant was interviewed with a carer present. There were no instances of one person interrupting, correcting or otherwise changing the response of another. However, it is possible that subtle influences arising from the relationship could have influenced participants’ responses when another was present. The qualitative sample included a young adult living at home, both working and stay-at-home mothers, a young adult with an intellectual disability and retired or semi-retired males and females, whose children had left home.

#### Qualitative findings

The qualitative findings facilitate a deeper understanding of the quantitative results from the program participants’ perspectives. Two key themes to emerge from the data were changes in food shopping behaviours and in social interactions at home through domestic cooking practices.

Participants reported purchasing a wider variety of fresh foods, such as fruit and vegetables, and less ‘packet’ and processed/prepared foods; many viewed this as a consequence of preparing more meals from scratch. Six months after the program, there were examples of participants shopping smarter, *“buying more to our list”* and growing their own vegetables and herbs.

*“I was stuffing the fatty things [in the trolley] and I wouldn’t change and try new stuff, which was costing me more money and now I’m trying all these new things, I might spend a bit more on fresh fruit and vegetables than what I used to but…. It’s a good thing [it] means we are not buying crap…”*

However, for a number of participants, shopping behaviour did not appear to change. Several retired older participants claimed they were “*set in their ways*” and had not made many changes to their food purchasing, nor noticed many distinct changes in their food preferences. Some older participants indicated that the effort involved and the cost of ingredients, prevented them from making, for example a curry paste from scratch, when they could either go out for a meal or purchase the prepared version. Premixed sauces, for example, were seen as “*getting close to authentic*” compared to what they were like in the past. For older participants, prepared meals continued to be an easy option for ease and convenience. However this attitude did not persist amongst younger participants, particularly those with children living at home. The decision to make a meal from scratch appeared to be more influenced by whom they were cooking for. Those with children or young adults residing at home were more likely to invest energy in providing a “*proper*” meal made from scratch and containing vegetables. There was limited discussion around the consumption of takeaway food. However there was discussion around the role that time plays when cooking at home and how increases in their cooking confidence and skills to prepare meals quickly from scratch may have contributed to a decrease in take-away expenditure.

As participants gained confidence in their cooking abilities and found enjoyment in attending the program, the benefits gained in the class were taken home and shared with others. Firstly, they described sharing their program experience with others, through sharing the knowledge they had acquired and the food they had prepared with friends, family and colleagues. For many, this brought about feelings of accomplishment and encouragement and interest from others that did not attend the program. Secondly, a small number of participants described changes after the program in their ability and confidence to prepare a meal for others. Some participants endeavoured to prepare “*fun”* meals with others, whether this was sharing the food they made in class, or preparing and serving a new meal like Jamie would on shared platters, or moving away from eating in front of the television to eating at the table. For many, sharing their program experience provided a positive experience that added to their cooking enjoyment and satisfaction.

All interview participants were unanimous about the importance of eating meals together with other people. There were reports of more social interactions in the home environment after attending the JMoF program, with many describing *“an opportunity to have family time cooking”.* Social cooking and eating as a family resulted in shared food enjoyment and special memories for some. There were reported changes to the family environment and in family interactions. It was commonly mentioned, that working as a household team to prepare meals was still occurring six months after finishing the program. Some participants reported talking more about food and cooking practices and meal planning than before doing the program. Some reported physical changes to the home environment.

*“We've made a bit more space and… we sort of moved some things around, so now when I’m cooking, the kids can sit up on the kitchen bench and we can still do reading or some of the homework so there’s still that interaction, whereas before we didn’t have it set up like that… I think it’s changed the dynamics, which we wouldn’t have bothered to change at all if we hadn’t come along [to the program]… It was like, well if the kids needed help I had to go and help them out. I couldn’t be cooking. The risotto needs to be stirred continuously. You can’t do that if every minute “can you help me with…” but now it’s like “yeah, I’m doing this”. Yeah but putting those changes into place…”*

## Discussion

This study which uses a longitudinal mixed method design including a wait-list control group, is the first to evaluate a JMoF program. The evaluation adds to the body of literature around cooking skills interventions which to date has been limited in showing effectiveness of impacts [34]. This study has demonstrated that the 10-week JMoF program has provided small but positive sustained effect on intervention participants’ attitudes, beliefs, knowledge and enjoyment around cooking and healthy eating. The strongest changes in attitudes concerned being able to prepare a meal in 30 minutes and able to prepare a meal from basics that was low in cost. This was also reflected in participants’ pride in being able to discuss and demonstrate their program experiences to others. There were also small improvements in eating at the dinner table, expenditure on take-away or fast foods, as well as self-perceived health and self-esteem. Attitudes and beliefs are understood to be good predictors of behaviour [35], so improved attitudes associated with participation in the program is a positive outcome. The confidence and skills gained by participants from attending the JMoF program gave them a fresh attitude to cooking, which in turn enabled them to review and change cooking and meal practices at home. The baseline values suggest that the evaluation participants started in the mid-range of cooking skills, attitudes and knowledge. So there is a possibility that the program captured people who may not have needed it and may attract people with at least some cooking skills which they wanted to improve. While each individually measured change was small, together they represent a move towards positive behaviour change.

Six months after finishing the program, JMoF participants were spending on average 4.15AUD less on take away/fast foods per week, which is consistent with the finding that participants were consuming less take away/fast food [24]. To put this into context the cost of a “Big Mac” in Australia according to the Big Mac index is approximately AUD5.15 [36]. Whilst participants’ overall weekly food and drink expenditure did not change, they were spending more on fruit and vegetables. This aligns with qualitative findings which indicated that there was a change in attitude around food spending with some participants favouring cooking from scratch using fresh ingredients rather than packaged convenience foods. This appeared to be a direct consequence of their improved cooking confidence and knowledge. This attitude shift was aligned with life stage, with younger participants and those who had children living at home more motivated to change. In summary, food expenditure changes appeared to be driven from a prioritisation of cooking from scratch in the act of providing a ‘proper’ meal to those living at home.

Another cooking skills intervention program conducted in Australia, The Food Cent$ program, aimed to improve diets and change food purchasing behaviours through developing budgeting, cooking and shopping skills and knowledge [5]. Through the provision of budgeting skills the program led to significant dietary behaviour changes - more vegetables consumed, decreased consumption of confectionary items and fewer purchases of cakes. The Can Cook Family programme in the UK [37] showed that participants increased their mean percentage weekly spend on fruit and vegetable expenditure by 2.55% after the program, which is similar to the estimated increase in the percentage (2.07% = 100 × ( (23.64/137.28) – (20.77/137.13)) six months after participation in the JMoF program. The Can Cook Family Programme also led to a reduction of takeaway meals purchased and indicated this was due to improvements in participants’ cooking confidence [37]. These two programs (Food Cent$ and Can Cook programs) however had small sample sizes and no control group. The JMoF program has a larger sample size and a control group and therefore makes a stronger case that cooking skills interventions have an impact on food purchasing behaviours and this can lead to healthier diets.

Results showed that over time the JMoF program led to an increase in eating at the table and an associated decrease in eating in front of the TV. These changes were also reflected in the qualitative findings. Participants, particularly those with children living at home, found more enjoyment from cooking and involving others in planning, cooking and sharing meals. There are a number of consequences arising from changes in social and mealtime behaviours. Firstly, social connectedness can be fostered through positive family relationships [38]. Cooking and eating together at home results in families spending more quality time together, thereby providing an opportunity for social support and improved family relationships. Increased family meal frequency and children’s involvement in cooking is also likely to improve the nutritional quality of a meal [6, 17]. Children’s involvement in the cooking process has been found to increase consumption of fruit and vegetables [17]. Participants’ greater confidence and enjoyment of cooking translated to more positive shared experiences of both preparing meals and eating together as a family.

An Australia wide study conducted in 2008 looking at family dinners found 77% of families ate together at mealtime five or more times per week [19]. To make a comparison to the JMoF program proportions showed that, at T3, approximately 72% of JMoF participants who had children living at home reported eating together 5 times per week. This had increased from approximately 66% at baseline. Direct comparison must be treated with caution because the JMOF sample represents greater social disadvantage, and geographical differences than the Huntley (2008) sample. However, it does indicate scope for further improvements.

Typically, evaluations of cooking skills interventions report on social outcomes in terms of social connections and support experienced through attendance of the program such as social support, friendship building and information sharing [8, 39, 40]. Keller et al. reported findings from a men’s cooking skills intervention in which qualitative findings suggested the program had a positive effect on participants’ sense of self-worth and connection with others [8]. Engler-Stringer and Berenbaum [40] explored social support developed through participation in collective kitchens through qualitative participant observations and interviews. Findings suggested that there were improvements in social isolation, increased social support, participation and sharing resources in their community as well as knowledge of where to find help [40]. There is limited evidence available on the social effect beyond the program. The JMoF evaluation offers new findings that highlight improved social interactions through domestic cooking within the home.

BMI did not show an overall effect either between groups or over time and this was not unexpected given that the program did not focus on weight reduction during its 10-week intervention. BMI is rarely reported or measured in evaluations of cooking skills interventions. One study that did report on children’s BMI after a five 90-minute parent/child cooking intervention addressing obesity revealed that BMI did not significantly change after program attendance [6]. Another intervention called Raising the Bar on Nutrition, aimed at food pantry clients, did show a significant reduction in BMI after a six week cooking program, however this study had low participant numbers (n = 54) and did not have a control group [41]. Hall et al. (2011) states that weight loss response is slow [42] and therefore it would not be expected in a program of this nature and duration. If the study had a T3 control, there is a possibility that results may have shown a difference between control and intervention groups, however speculating this is not possible within the current study design and beyond the data presented.

While this study has strengths, it is not without its limitations. Qualitative findings are based on a purposive sub-sample of JMoF participants willing to be interviewed. It successfully captured a range of perspectives but the findings, shaped by their personal insights and experiences, may not be translatable to all JMoF participants. Both quantitative and qualitative findings may possess an element of social desirability bias which is often common in self-report measures and interview data [43]. On the other hand such measures are commonly used, until alternatives are devised. Another issue is the possibility of biases related to “Readiness to change” that may impact on the comparison of wait-list control groups with intervention groups, a recent paper [44] has attempted to investigate this (in the context of problem drinking). If the highly ready are forced to wait they might start changing and the effect of the intervention is underestimated. Accidental confounding of readiness-to-change with the control group is then a problem. Randomization would reduce confounding however randomisation within real life settings often poses barriers to the intervention [45]. A randomised control would have also been unsuitable for participation in the JMoF program because participants often attend at a convenient time with family and friends. The low literacy level of some participants may also have impacted on results, however literacy of participants were not measured in this evaluation, which may be required in future studies. While the questionnaire used clear simple language and was piloted within Ipswich, there may be misinterpretations of questions within the population.

Results presented in this paper suggest there were sustained improvements in attitudes, knowledge and purchasing behaviours around the consumption and preparation of vegetables after the program. The program had a sustained impact on participants’ cooking enjoyment and satisfaction, which linked heavily to improved social interaction around cooking and meal consumption within the family home. Many changes resulting from the program were statistically significant but small and sustained. The program implementers need to explore ways in which the participant benefits gained as a consequence of the program can be sustained over time.

## Conclusions

This study is the first rigorous evaluation of the JMoF program that incorporates a control group, a mixed methods design, and a follow-up period. Results showed multiple improvements in participants’ food and cooking attitudes and knowledge, food purchasing behaviours and social interactions within the home environment, which were sustained six months after the program, adding to the limited evidence of the wider impacts of cooking skills interventions.

## Electronic supplementary material

Additional file 1: **Secondary outcome measures between intervention and control group at baseline and follow up adjusted for age, gender, employment and combined.** Additional analyses to show adjusted results for intervention and control at baseline and follow up. (PDF 143 KB)
