# Supplementary material for: Wider impacts of a 10-week community cooking skills program - Jamie’s Ministry of Food, Australia
Source: BMC Public Health. 2014 Dec 12;14:1161. doi: 10.1186/1471-2458-14-1161 (PMC4295497; doi:10.1186/1471-2458-14-1161)
Supplement: Supplementary file 1 — Additional file 1: Secondary outcome measures between intervention and control group at baseline and follow up adjusted for age, gender, employment and combined. Additional analyses to show adjusted results for intervention and control at baseline and follow up. (PDF 143 KB) [file 12889_2014_7344_MOESM1_ESM.pdf]

Additional File 1

Secondary outcome measures between intervention and control group at baseline and follow up adjusted for age, gender, employment and combined[1]

Outcome measure	Intervention			Control			Difference between groups in changes over time (interaction effect)[3]
	baseline (T1) mean (S.E)[2]	follow up (T2) mean (S.E)	change from baseline(T2-T1) mean (S.E) *P value*	baseline (T1) mean (S.E)[2]	follow up (T2) mean (S.E)	change from baseline(T2-T1) mean (S.E) *P value*	*P value*
**Food Purchasing behaviours and attitudes**							
Total weekly food and drink expenditure (AUD)[4]	137.16 (2.72)	135.60 (3.15)	-1.56 (2.46) P = 0.53	147.34 (4.68)	151.68 (5.20)	4.33 (3.96) P=0.27	P = 0.21
Age	136.98 (2.63)	136.42 (2.99)	-0.56 (2.44) P = 0.82	144.15 (4.61)	148.26 (5.01)	4.11 (4.00) P=0.30	P = 0.32
Gender	137.25 (2.75)	135.40 (3.14)	-1.85 (2.46) P = 0.45	149.03 (4.79)	151.97 (5.22)	2.94 (3.99) P=0.46	P = 0.31
Employment	137.61 (2.63)	136.46 (2.98)	-1.14 (2.45) P = 0.64	144.82 (4.54)	148.52 (4.89)	3.70 (3.94) P=0.35	P = 0.30
Age, gender, employment	136.84 (2.60)	136.31 (2.95)	-0.53 (2.44) P = 0.83	144.96 (4.56)	148.81 (4.95)	3.85 (4.00) P=0.34	P = 0.35
Total weekly fruit and veg expenditure (AUD)[4]	20.77 (0.61)	23.28 (0.73)	2.50 (0.63) P<0.001	21.70 (1.06)	22.24 (1.20)	0.53 (1.01) P=0.60	P = 0.10
Age	20.77 (0.60)	23.53 (9.73)	2.76 (0.63) P<0.001	21.28 (1.06)	21.44 (1.21)	0.16 (1.04) P=0.88	P = 0.03
Gender	20.73 (0.61)	23.31 (0.74)	2.58 (0.63) P<0.001	21.92 (1.07)	22.20 (1.23)	0.28 (1.02) P=0.79	P = 0.06
Employment	20.75 (0.61)	23.56 (0.73)	2.81 (0.63) P<0.001	21.33 (1.05)	21.63 (1.20)	0.29 (1.01) P=0.77	P = 0.04
Age, gender, employment	20.66 (0.60)	23.53 (0.73)	2.87 (0.64) P<0.001	21.37 (1.06)	21.66 (1.22)	0.28 (1.04) P=0.79	P = 0.03
Total weekly take away/fast food expenditure (AUD)[4]	13.17 (0.59)	9.86 (0.69)	-3.31 (0.55) P<0.001	12.395 (1.01)	12.05 (1.13)	-0.34 (0.87) P=0.70	P = 0.004
Age	13.17 (0.58)	9.99 (0.60)	-3.19 (0.54) P<0.001	11.50 (1.01)	11.26 (1.0)	-0.24 (0.88) P=0.78	P = 0.005
Gender	13.28 (0.62)	9.65 (0.62)	-3.63 (0.53) P<0.001	12.47 (1.07)	11.73 (1.03)	-0.73 (0.86) P=0.40	P = 0.004
Employment	13.22 (0.58)	9.79 (0.59)	-3.43 (0.54)P<0.001	11.78 (1.01)	11.33 (0.97)	-0.45 (0.87) P=0.60	P = 0.004
Age, gender, employment	13.15 (0.57)	9.93 (0.59)	-3.22 (0.54) P<0.001	11.56 (1.01)	11.07 (0.98)	-0.48 (0.88) P=0.57	P = 0.01
I can Prepare a meal from basics that is low in Price[5]	2.99 (0.03)	3.41 (0.04)	0.41 (0.04) P<0.001	3.00 (0.05)	2.97 (0.06)	-0.02 (0.06) P=0.71	P < 0.001
Age	3.00 (0.03)	3.40 (0.03)	0.40 (0.04) P<0.001	3.01 (0.05)	2.98 (0.05)	-0.02 (0.06) P=0.70	P < 0.001
Gender	3.00 (0.03)	3.41 (0.03)	0.41 (0.04) P<0.001	2.99 (0.05)	2.97 (0.05)	-0.03 (0.06) P=0.67	P < 0.001
Employment	3.00 (0.03)	3.40 (0.03)	0.40 (0.04) P<0.001	3.01 (0.05)	2.98 (0.05)	-0.03 (0.06) P=0.63	P < 0.001
Age, gender, employment	3.00 (0.03)	3.40 (0.03)	0.40 (0.04) P<0.001	3.00 (0.05)	2.97 (0.05)	-0.03 (0.06) P=0.68	P < 0.001
Buying more fruit/vegetables would not be difficult on my budget[5a]	2.85 (0.03)	2.93 (0.04)	0.08 (0.04) P = 0.06	2.85 (0.06)	2.89 (0.07)	0.04 (0.07) P=0.59	P = 0.60
Age	2.86 (0.03)	2.93 (0.04)	0.07 (0.04) P = 0.10	2.86 (0.06)	2.87 (0.07)	0.01 (0.07) P=0.94	P = 0.43
Gender	2.86 (0.03)	2.93 (0.04)	0.06 (0.04) P = 0.15	2.87 (0.06)	2.90 (0.07)	0.04 (0.07) P=0.60	P = 0.75
Employment	2.87 (0.03)	2.93 (0.04)	0.06 (0.04) P = 0.17	2.85 (0.06)	2.89 (0.07)	0.03 (0.07) P=0.65	P = 0.74
Age, gender, employment	2.87 (0.03)	2.93 (0.04)	0 .06 (0.05) P = 0.16	2.85 (0.06)	2.86 (0.07)	0.01 (0.07) P=0.84	P = 0.58
Fruit and vegetables are cheaper when they are in season[5]	3.42 (0.02)	3.62 (0.03)	0.21 (0.03) P<0.001	3.43 (0.04)	3.50 (0.05)	0.07 (0.06) P=0.21	P = 0.04
Age	3.41 (0.03)	3.62 (0.03)	0.21 (0.03) P<0.001	3.44 (0.05)	3.51 (0.05)	0.07 (0.06) P=0.23	P = 0.03
Gender	3.41 (0.03)	3.62 (0.03)	0.21 (0.03) P<0.001	3.43 (0.05)	3.50 (0.05)	0.07 (0.06) P=0.19	P = 0.03
Employment	3.41 (0.03)	3.62 (0.03)	0.21 (0.03) P<0.001	3.44 (0.05)	3.50 (0.05)	0.07 (0.06) P=0.24	P = 0.03
Age, gender, employment	3.41 (0.03)	3.62 (0.03)	0.21 (0.03) P<0.001	3.42 (0.05)	3.50 (0.05)	0.07 (0.06) P=0.19	P = 0.05
**Cooking and healthy eating knowledge, attitudes beliefs and**							

Outcome measure	Intervention			Control			Difference between groups in changes over time (interaction effect)[3] *P value*
	baseline (T1) mean (S.E)[2]	follow up (T2) mean (S.E)	change from baseline(T2-T1) mean (S.E) *P value*	baseline (T1) mean (S.E)[2]	follow up (T2) mean (S.E)	change from baseline(T2-T1) mean (S.E) *P value*	
**behaviours**							
I can Put together a healthy meal from scratch in 30 minutes[5]	2.85 (0.031)	3.30 (0.04)	0.45 (0.04) P<0.001	2.85 (0.05)	2.89 (0.06)	0.03 (0.06) P=0.61	P < 0.001
Age	2.86 (0.03)	3.30 (0.03)	0.44 (0.04) P<0.001	2.87 (0.06)	2.89 (0.06)	0.02 (0.06) P=0.76	P < 0.001
Gender	2.86 (0.03)	3.30 (0.03)	0.44 (0.04) P<0.001	2.85 (0.06)	2.87 (0.06)	0.02 (0.06) P=0.75	P < 0.001
Employment	2.86 (0.03)	3.29 (0.03)	0.43 (0.04) P<0.001	2.86 (0.06)	2.88 (0.06)	0.02 (0.06) P=0.72	P < 0.001
Age, gender, employment	2.87 (0.03)	3.30 (0.03)	0.44 (0.04) P<0.001	2.86 (0.06)	2.88 (0.06)	0.01 (0.06) P=0.82	P < 0.001
I find it easy to change my eating habits [5]	2.52 (0.03)	2.71 (0.04)	0.19 (0.04) P<0.001	2.52 (0.05)	2.53 (0.06)	0.01 (0.06) P=0.82	P = 0.02
Age	2.52 (0.03)	2.71 (0.04)	0.19 (0.04) P<0.001	2.53 (0.05)	2.54 (0.06)	0.01 (0.07) P=0.89	P = 0.02
Gender	2.51 (0.03)	2.70 (0.04)	0.19 (0.04) P<0.001	2.53 (0.05)	2.55 (0.06)	0.02 (0.06) P=0.71	P = 0.03
Employment	2.51 (0.03)	2.70 (0.04)	0.19 (0.04) P<0.001	2.53 (0.05)	2.55 (0.06)	0.01 (0.06) P=0.82	P = 0.02
Age, gender, employment	2.51 (0.03)	2.71 (0.04)	0.20 (0.04) P<0.001	2.54 (0.05)	2.56 (0.06)	0.02 (0.07) P=0.78	P = 0.02
Vegetables can be tasty foods[5]	3.54 (0.02)	3.69 (0.03)	0.15 (0.03) P<0.001	3.53 (0.04)	3.51 (0.05)	-0.02 (0.05) P=0.74	P = 0.01
Age	3.54 (0.02)	3.68 (0.03)	0.14 (0.03) P<0.001	3.54 (0.04)	3.51 (0.05)	-0.03 (0.05) P=0.52	P = 0.003
Gender	3.54 (0.02)	3.69 (0.03)	0.15 (0.03) P<0.001	3.53 (0.04)	3.51 (0.04)	-0.02 (0.05) P=0.75	P = 0.01
Employment	3.54 (0.02)	3.69 (0.03)	0.15 (0.03) P<0.001	3.53 (0.04)	3.51 (0.05)	-0.01 (0.05) P=0.77	P = 0.01
Age, gender, employment	3.54 (0.02)	3.69 (0.03)	0.14 (0.03) P<0.001	3.54 (0.04)	3.51 (0.05)	-0.03 (0.05) P=0.57	P = 0.003
I eat enough fruit and vegetables[5]	2.66 (0.03)	3.00 (0.04)	0.34 (0.04) P<0.001	2.66 (0.06)	2.68 (0.07)	0.02 (0.06) P=0.71	P < 0.001
Age	2.66 (0.03)	2.98 (0.04)	0.32 (0.04) P<0.001	2.70 (0.06)	2.70 (0.06)	0.00 (0.06) P=0.96	P < 0.001
Gender	2.65 (0.03)	2.99 (0.04)	0.34 (0.04) P<0.001	2.68 (0.06)	2.69 (0.06)	0.01 (0.06) P=0.83	P < 0.001
Employment	2.67 (0.03)	3.00 (0.04)	0.33 (0.04) P<0.001	2.68 (0.06)	2.69 (0.06)	0.01 (0.06) P=0.89	P < 0.001
Age, gender, employment	2.67 (0.03)	2.99 (0.04)	0.32 (0.04) P<0.001	2.70 (0.06)	2.70 (0.06)	0.01 (0.06) P=0.93	P < 0.001
My lifestyle does not Prevent me eating a healthy diet[5a]	3.11 (0.03)	3.33 (0.04)	0.22 (0.04) P<0.001	3.04 (0.05)	3.12 (0.06)	0.08 (0.06) P=0.17	P = 0.07
Age	3.11 (0.03)	3.32 (0.04)	0.21 (0.04) P<0.001	3.06 (0.05)	3.14 (0.06)	0.07 (0.06) P=0.17	P = 0.10
Gender	3.11 (0.03)	3.33 (0.04)	0.21 (0.04) P<0.001	3.04 (0.05)	3.12 (0.06)	0.08 (0.06) P=0.19	P = 0.07
Employment	3.12 (0.03)	3.32 (0.04)	0.20 (0.04) P<0.001	3.05 (0.05)	3.13 (0.06)	0.08 (0.06) P=0.18	P = 0.09
Age, gender, employment	3.17 (0.03)	3.32 (0.04)	0.21 (0.04) P<0.001	3.06 (0.05)	3.14 (0.06)	0.08 (0.06) P=0.21	P = 0.08
**Cooking enjoyment and satisfaction**							
I enjoy cooking[5]	3.05 (0.03)	3.33 (0.04)	0.28 (0.03) P<0.001	3.12 (0.05)	3.17 (0.06)	0.06 (0.05) P=0.28	P = 0.001
Age	3.05 (0.03)	3.34 (0.03)	0.29 (0.03) P<0.001	3.08 (0.06)	3.15 (0.06)	0.06 (0.05) P= 0.24	P<0.001
Gender	3.06 (0.03)	3.33 (0.03)	0 .27 (0.03) P<0.001	3.12 (0.06)	3.16 (0.06)	0.04 (0.05) P=0.41	P<0.001
Employment	3.06 (0.03)	3.34 (0.03)	0.28 (0.03) P<0.001	3.10 (0.06)	3.16 (0.06)	0.06 (0.05) P=0.27	P<0.001
Age, gender, employment	3.06 (0.03)	3.35 (0.03)	0.30 (0.03) P<0.001	3.09 (0.06)	3.14 (0.06)	0.05 (0.05) P=0.32	P<0.001
I get a lot of satisfaction from cooking my meals[5]	2.96 (0.03)	3.31 (0.04)	0.35 (0.03) P<0.001	3.02 (0.05)	3.05 (0.06)	0.03 (0.05) P=0.60	P < 0.001
Age	2.97 (0.03)	3.33 (0.03)	0.36 (0.03) P<0.001	3.00 (0.06)	3.02 (0.06)	0.02 (0.05) P=0.70	P<0.001
Gender	2.97 (0.03)	3.32 (0.04)	0.35 (0.03) P<0.001	3.03 (0.06)	3.03 (0.06)	0.01 (0.05) P=0.89	P<0.001
Employment	2.97 (0.03)	3.32 (0.04)	0.35 (0.03) P<0.001	3.00 (0.06)	3.03 (0.06)	0.02 (0.05) P=0.68	P<0.001
Age, gender, employment	2.97 (0.03)	3.33 (0.03)	0.36 (0.03) P<0.001	3.01 (0.06)	3.02 (0.06)	0.01 (0.05) P=0.83	P<0.001
I enjoy cooking for others[5]	3.01 (0.03)	3.27 (0.04)	0.26 (0.03) P<0.001	3.09 (0.06)	3.16 (0.07)	0.07 (0.06) P=0.22	P = 0.004

Outcome measure	Intervention			Control			Difference between groups in changes over time (interaction effect)[3]
	baseline (T1) mean (S.E)[2]	follow up (T2) mean (S.E)	change from baseline(T2-T1) mean (S.E) *P value*	baseline (T1) mean (S.E)[2]	follow up (T2) mean (S.E)	change from baseline(T2-T1) mean (S.E) *P value*	*P value*
Age	3.01 (0.03)	3.28 (0.04)	0.27 (0.03) P<0.001	3.06 (0.06)	3.13 (0.06)	0.07 (0.06) P=0.19	P = 0.003
Gender	3.02 (0.03)	3.27 (0.04)	0.25 (0.03) P<0.001	3.09 (0.06)	3.14 (0.06)	0 .06 (0.05) P=0.31	P = 0.003
Employment	3.01 (0.03)	3.27 (0.04)	0.26 (0.03) P<0.001	3.07 (0.06)	3.14 (0.06)	0.07 (0.05) P=0.20	P = 0.004
Age, gender, employment	3.02 (0.03)	3.28 (0.04)	0.27 (0.03) P<0.001	3.06 (0.06)	3.12 (0.06)	0.06 (0.06) P=0.26	P = 0.002
I *enjoy eating a meal with others*[5]	3.51 (0.02)	3.60 (0.03)	0.09 (0.03) P = 0.01	3.47 (0.39)	3.55 (0.05)	0.07 (0.05) P=0.16	P = 0.81
Age	3.51 (0.02)	3.60 (0.03)	0.09 (0.03) P=0.004	3.47 (0.04)	3.54 (0.04)	0.07 (0.05) P=0.17	P = 0.75
Gender	3.52 (0.02)	3.60 (0.03)	0.08 (0.03) P = 0.01	3.47 (0.04)	3.54 (0.04)	0.07 (0.05) P=0.16	P = 0.83
Employment	3.51 (0.02)	3.60 (0.02)	0.09 (0.03) P=0.004	3.47 (0.04)	3.54 (0.04)	0.07 (0.05) P=0.16	P = 0.76
Age, gender, employment	3.52 (0.02)	3.61 (0.03)	0.09 (0.03) P = 0.01	3.47 (0.04)	3.53 (0.05)	0.07 (0.05) P=0.17	P = 0.75
**Social eating**							
Frequency of eating together at home with others[6]	3.94 (0.07)	4.20 (0.08)	0.24 (0.07) P<0.001	3.97 (0.11)	4.02 (0.13)	0.06 (0.11) P=0.61	P = 0.13
Age	3.93 (0.07)	4.20 (0.08)	0.27 (0.07) P<0.001	3.97 (0.12)	4.08 (0.12)	0.11 (0.11) P=0.31	P = 0.23
Gender	3.93 (0.07)	4.19 (0.08)	0.27 (0.07) P<0.001	4.00 (0.12)	4.07 (0.12)	0.07 (0.12) P=0.51	P = 0.13
Employment	3.95 (0.07)	4.21 (0.08)	0.27 (0.07) P<0.001	3.98 (0.12)	4.08 (0.12)	0.11 (0.11) P=0.33	P = 0.20
Age, gender, employment	3.95 (0.07)	4.21 (0.08)	0.27 (0.07) P<0.001	3.98 (0.12)	4.06 (0.12)	0.08 (0.11) P=0.45	P = 0.15
Frequency of eating dinner in front of the television[6]	2.69 (0.08)	2.50 (0.09)	-0.19 (0.07) P = 0.01	2.51 (0.14)	2.52 (0.15)	0.00 (0.11) P=0.99	P = 0.17
Age	2.69 (0.08)	2.49 (0.09)	-0.19 (0.07) P = 0.01	2.49 (0.14)	2.45 (0.15)	0.01 (0.12) P=0.92	P = 0.14
Gender	2.69 (0.08)	2.52 (0.09)	-0.18 (0.07) P = 0.01	2.48 (0.14)	2.48 (0.15)	0.00 (0.12) P=0.98	P = 0.18
Employment	2.69 (0.08)	2.52 (0.09)	-0.17 (0.07) P = 0.02	2.46 (0.14)	2.48 (0.15)	0.01 (0.12) P=0.90	P = 0.17
Age, gender, employment	2.70 (0.08)	2.51 (0.09)	-0.19 (0.07) P = 0.01	2.44 (0.14)	2.44 (0.15)	0.00 (0.12) P=0.97	P = 0.16
Frequency of eating dinner at a dinner table[6]	3.12 (0.08)	3.40 (0.09)	0.29 (0.06) P<0.001	3.11 (0.13)	3.09 (0.14)	-0.02 (0.10) P=0.86	P = 0.01
Age	3.12 (0.08)	3.40 (0.08)	0.28 (0.06) P<0.001	3.14 (0.14)	3.17 (0.14)	0.03 (0.10) P=0.76	P = 0.03
Gender	3.12 (0.08)	3.40 (0.08)	0.28 (0.06) P<0.001	3.13 (0.14)	3.12 (0.14)	-0.01 (0.10) P=0.89	P = 0.01
Employment	3.13 (0.08)	3.41 (0.08)	0.28 (0.06) P<0.001	3.15 (0.13)	3.14 (0.14)	-0.01 (0.10) P=0.89	P = 0.01
Age, gender, employment	3.13 (0.08)	3.41 (0.08)	0.28 (0.06) P<0.001	3.17 (0.14)	3.20 (0.14)	0.02 (0.10) P=0.82	P = 0.03
**Health and emotional well-being**							
Global self-esteem score[7]	20.88 (0.22)	22.60 (0.25)	1.73 (0.20) P<0.001	20.46 (0.37)	21.02 (0.42)	0.56 (0.32) P=0.09	P = 0.002
Age	20.90 (0.22)	22.58 (0.25)	1.68 (0.20) P<0.001	20.53 (0.38)	21.07 (0.42)	0.54 (0.33) P=0.10	P = 0.003
Gender	20.85 (0.22)	22.58 (0.25)	1.73 (0.20) P<0.001	20.50 (0.38)	21.12 (0.42)	0.62 (0.32) P=0.06	P = 0.003
Employment	20.94 (0.22)	22.65 (0.25)	1.71 (0.20) P<0.001	20.47 (0.37)	21.05 (0.41)	0.58 (0.32) P=0.07	P = 0.003
Age, gender, employment	20.91 (0.21)	22.62 (0.25)	1.71 (0.20) P<0.001	20.60 (0.37)	21.17 (0.42)	0.58 (0.33) P=0.08	P = 0.004
General Health[8]	2.77 (0.04)	3.11 (0.04)	0.34 (0.04) P<0.001	2.80 (0.06)	2.86 (0.07)	0.06 (0.06) P=0.34	P < 0.001
Age	2.77 (0.04)	3.11 (0.04)	0.34 (0.04) P<0.001	2.83 (0.06)	2.90 (0.07)	0.07 (0.06) P=0.26	P < 0.001
Gender	2.76 (0.04)	3.10 (0.04)	0.34 (0.04) P<0.001	2.82 (0.07)	2.89 (0.07)	0.07 (0.06) P=0.24	P < 0.001
Employment	2.77 (0.04)	3.12 (0.04)	0.34 (0.04) P<0.001	2.83 (0.06)	2.89 (0.07)	0.06 (0.06) P=0.33	P < 0.001
Age, gender, employment	2.77 (0.04)	3.11 (0.04)	0.34 (0.04) P<0.001	2.84 (0.07)	2.92 (0.07)	0.07 (0.06) P=0.22	P < 0.001
Body Mass Index (BMI)	28.86 (0.27)	28.78 (0.28)	-0.09 (0.13) P = 0.49	29.71 (0.46)	29.70 (0.47)	-0.02 (0.20) P=0.94	P = 0.76

Outcome measure	Intervention			Control			Difference between groups in changes over time (interaction effect)[3]
	baseline (T1) mean (S.E)[2]	follow up (T2) mean (S.E)	change from baseline(T2-T1) mean (S.E) *P value*	baseline (T1) mean (S.E)[2]	follow up (T2) mean (S.E)	change from baseline(T2-T1) mean (S.E) *P value*	*P value*
Age	28.86 (0.27)	28.77 (0.29)	-0.09 (0.13) P = 0.49	29.50 (0.47)	29.47 (0.49)	-0.04 (0.21) P=0.86	P = 0.83
Gender	28.83 (0.27)	28.74 (0.28)	-0.09 (0.13) P = 0.49	29.66 (0.46)	29.63 (0.48)	-0.03 (0.20) P=0.90	P = 0.80
Employment	28.90 (0.27)	28.80 (0.29)	-0.10 (0.13) P = 0.42	29.56 (0.46)	29.53 (0.48)	-0.03 (0.20) P=0.90	P = 0.75
Age, gender, employment	28.85 (0.28)	28.75 (0.29)	-0.10 (0.13) P = 0.43	29.54 (0.47)	29.50 (0.49)	-0.03 (0.21) P=0.87	P = 0.79

[1]Outcomes within each group and over time were determined by a mixed linear model for repeated measures using all available data at each time Point using STATA (version 12.0).
[2]Baseline values were not significantly different between groups (independent t tests)
[3]A significant group and time interaction effect denotes that the response over time differed between groups (*P=0.05)*
[4]Expenditure data was collected in Australian dollars (AUD) on a 7-Point scale which was analyse by its midpoints. [5] Mean Predicted score indicating level of agreement with statement from a Likert Scale (*1=strongly disagree, 2=somewhat disagree, 3=somewhat agree, 4=strongly agree),* [a] Score assignment was reversed.
[6]Mean frequency for a typical week was collected on a 6 or 7-Point scale which was analyse by its midpoint, with the maximum category being five or more times Per week.
[7] Rosenberg's global self-esteem score *(Low self-esteem= 0-14, Normal self-esteem=15-25 and High self-esteem =16-30)*
[8] Perceived general health (*Poor=1, fair=2, good=3, very good=4, excellent=5)*
